# Effectiveness of a Mobile Phone-Delivered Multiple Health Behavior Change Intervention (LIFE4YOUth) in Adolescents: Randomized Controlled Trial

**DOI:** 10.2196/69425

**Published:** 2025-04-22

**Authors:** Anna Seiterö, Pontus Henriksson, Kristin Thomas, Hanna Henriksson, Marie Löf, Marcus Bendtsen, Ulrika Müssener

**Affiliations:** 1 Department of Health, Medicine and Caring Sciences Linköping University Linköping Sweden; 2 Department of Medicine, Huddinge Karolinska Institutet Stockholm Sweden

**Keywords:** mHealth, multiple behavior, high school students, digital behavior change intervention, public health, telemedicine, randomized controlled trial

## Abstract

**Background:**

Although mobile health (mHealth) interventions have demonstrated effectiveness in modifying 1 or 2 health-risk behaviors at a time, there is a knowledge gap regarding the effects of stand-alone mHealth interventions on multiple health risk behaviors.

**Objective:**

This study aimed to estimate the 2- and 4-month effectiveness of an mHealth intervention (LIFE4YOUth) targeting alcohol consumption, diet, physical activity, and smoking among Swedish high school students, compared with a waiting-list control condition.

**Methods:**

A 2-arm parallel group, single-blind randomized controlled trial (1:1) was conducted from September 2020 to June 2023. Eligibility criteria included nonadherence to guidelines related to the primary outcomes, such as weekly alcohol consumption (standard drinks), monthly frequency of heavy episodic drinking (ie, ≥4 standard drinks), daily intake of fruit and vegetables (100-g portions), weekly consumption of sugary drinks (33-cL servings), weekly duration of moderate to vigorous physical activity (minutes), and 4-week point prevalence of smoking abstinence. The intervention group had 16 weeks of access to LIFE4YOUth, a fully automated intervention including recurring screening, text message services, and a web-based dashboard. Intention-to-treat analysis was conducted on available and imputed 2- and 4-month self-reported data from participants at risk for each outcome respectively, at baseline. Effects were estimated using multilevel models with adaptive intercepts (per individual) and time by group interactions, adjusted for baseline age, sex, household economy, and self-perceived importance, confidence, and know-how to change behaviors. Bayesian inference with standard (half-)normal priors and null-hypothesis testing was used to estimate the parameters of statistical models.

**Results:**

In total, 756 students (aged 15-20, mean 17.1, SD 1.2 years; 69%, 520/756 females; 31%, 236/756 males) from high schools across Sweden participated in the trial. Follow-up surveys were completed by 71% (539/756) of participants at 2 months and 57% (431/756) of participants at 4 months. Most participants in the intervention group (219/377, 58%) engaged with the intervention at least once. At 2 months, results indicated positive effects in the intervention group, with complete case data indicating median between-group differences in fruit and vegetable consumption (0.32 portions per day, 95% CI 0.13-0.52), physical activity (50 minutes per week, 95% CI –0.2 to 99.7), and incidence rate ratio for heavy episodic drinking (0.77, 95% CI 0.55-1.07). The odds ratio for smoking abstinence (1.09, 95% CI 0.34-3.64), incidence rate ratio for weekly alcohol consumption (0.69, 95% CI 0.27-1.83), and the number of sugary drinks consumed weekly (0.89, 95% CI 0.73-1.1) indicated inconclusive evidence for effects due to uncertainty in the estimates. At 4 months, a remaining effect was observed on physical activity only.

**Conclusions:**

Although underpowered, our findings suggest modest short-term effects of the LIFE4YOUth intervention, primarily on physical activity and fruit and vegetable consumption. Our results provide inconclusive evidence regarding weekly alcohol consumption and smoking abstinence.

**Trial Registration:**

ISRCTN Registry ISRCTN34468623; https://doi.org/10.1186/ISRCTN34468623

## Introduction

Excessive alcohol consumption, unhealthy diets, physical inactivity, and smoking are major contributors to premature mortality and morbidity through noncommunicable diseases [1-4]. Health behaviors founded early in life may have long-lasting effects on physical and mental health [5-8]. Thus, it is concerning that only 30% of adolescents globally are meeting the recommendation of 60 minutes of moderate-to-vigorous physical activity (MVPA) per day [9], and that the proportion of adolescents who drink sugary drinks daily is higher than those who consume fruit daily [10]. Also, it is well-known that risk behaviors such as alcohol consumption and smoking are typically initiated during adolescence [11,12]. Altogether, interventions that target adolescents’ health behaviors are motivated.

Furthermore, engaging in multiple risk behaviors has a multiplicative effect on health, rather than an additive effect based on the sum of the risks of individual behaviors [13]. Global data indicate that about 10% of adolescents can be considered as overall unhealthy due to engagement in multiple risk behaviors [14], while 2 out of 3 are engaged in combinations of health-promoting and health-risk behaviors that influence their overall health [14,15]. Multiple behavior-change interventions, which address the interrelationships among 2 or more health behaviors, may be a cost-effective way to reduce risk behaviors associated with NCDs [16-18].

Schools provide a powerful setting for health promotion and disease prevention among adolescents. Several systematic reviews suggest that school-based programs can effectively influence risk behaviors associated with NCDs [19-22]. However, the literature also highlights a concern that school-based interventions relying on school staff tend to become short-term projects due to barriers that impede long-term implementation [23,24]. Factors such as lack of time, limited support, or high turnover among staff and principals involved in delivering interventions can threaten both the effectiveness and sustainability of school-based interventions. As a result, sustainable implementation may be better supported through interventions that are not dependent on school staff.

Mobile health (mHealth) interventions, including SMS text messages and apps [25], have shown promise for delivering health interventions to adolescents [26,27]. Evidence indicates a potential for effect of mHealth interventions on physical activity [28-30], diet, [31-33] alcohol consumption, [34] and smoking cessation [35]. These interventions can be delivered either independently (ie, stand-alone interventions) or as part of multicomponent programs. Multicomponent programs targeting various risk behaviors [36-40], often include teacher-led educational modules, face-to-face counseling, or activities designed to enhance school staffs’ ability to promote students’ health and decision-making. For example, Champion et al [40] evaluated the efficacy of a school-based intervention (Health4Life) targeting 6 key lifestyle risk behaviors among adolescents. Their computer-based intervention included an mHealth component alongside structured teacher-led lessons. In contrast to multicomponent interventions, stand-alone mHealth interventions complement other school-based health interventions, but are not necessarily tied to them and do not rely on school staff for delivery.

So far, evidence-based stand-alone mHealth interventions predominantly focus on a limited number of health behaviors at a time. For example, interventions often target physical activity and diet [41-45], or various types of substance use, including alcohol consumption, cigarette smoking, and cannabis use [46-50]. Indeed, only 1 out of 16 school-based interventions using technology to target multiple health risk behaviors, which were included in a comprehensive systematic review [51], focused on the 4 major risk behaviors (ie, alcohol consumption, unhealthy diets, physical inactivity, and smoking) for NCDs concurrently. That was a web-based multicomponent intervention supplemented with text messages, conducted between 2009 and 2012 [52]. Thus, a knowledge gap remains regarding the potential of stand-alone interventions targeting the 4 major risks behaviors for NCDs. This study addressed this gap by investigating the effectiveness of a stand-alone mHealth intervention (LIFE4YOUth) that concurrently targets alcohol consumption, unhealthy diet, physical inactivity, and cigarette smoking among Swedish high school students.

## Methods

### Design

This study was part of the MoBILE (Mobile health Multiple lifestyle Behavior Interventions across the LifEspan) research program [53]. The research program was initiated in 2018 to enhance knowledge of the effectiveness of digital multiple health behavior change interventions across various populations across the lifespan. This specific study referred to Swedish high school students.

A 2-arm parallel group (1:1) single-blind randomized controlled trial was used to estimate the 2- and 4-month effectiveness of the LIFE4YOUth intervention on high school students’ health risk behaviors compared with a waiting-list control group.

### Recruitment

A total of 403 high schools across Sweden participated in the recruitment of participants between September 2020 and June 2023. A public list of high schools in Sweden, provided by the Swedish National Agency for Education, was used to recruit schools. Invitations, containing information about the project, were sent by email to the head of each school. The initial invitation was sent to approximately 1250 recipients in February 2020. In addition, to recruit schools, project information was disseminated through professional networks (eg, school nurses and physical education teachers) through social media (eg, Facebook), articles in printed professional magazines, and poster presentations at national conferences targeting school professionals. Online seminars were regularly organized to provide school staff with information and support.

All schools that agreed to promote the project to their students were sent digital and printed posters (A3 format) and leaflets (A5 format) to distribute on bulletin boards, tables, and digital screens in public areas within the schools. In addition, a short video presentation was provided to facilitate teachers to briefly introduce the study during lecture hours. The participating schools were reminded about the project through email approximately twice per year.

Once COVID-19 restrictions eased in March 2022, the recruitment strategy was expanded to include in-person school visits conducted by members of the research team. Approximately 40 school visits were organized in collaboration with school staff. These included information sessions in classrooms, attendance at public areas such as entrance halls using a roll-up banner to increase visibility, and participation in whole-school activities focused on health. In addition, a student ambassador program was launched, where participants who recruited 5 additional participants were given a gift card worth approximately US $9. Nearly 100 students registered their interest in becoming ambassadors, and 10 successfully recruited 5 peers, earning a gift card.

To participate, high school students had to send a text message for trial information and actively confirm their consent. Eligibility was assessed through a web-based baseline questionnaire (Multimedia Appendix 1). Students meeting at least one of the following criteria based on their previous week’s health behaviors were included: ≥10 standard drinks of alcohol (12 g of pure alcohol); <500 g of fruit and vegetables (FV) on average per day; 2 or more units of sugary drinks (33 cL); <420 minutes of MVPA; 1 or more smoked cigarette. For alcohol, 4 or more standard drinks on a single occasion at least once in the past month was also a criterion. Examples were used to help students interpret the threshold amounts. For example, participants were informed that 100 g of FV is roughly equivalent to an average sized banana or one large apple, or vegetables to an average handful. The sugary drink criteria were modified because the questionnaire did not offer a response option for ≥3 units per week, which was originally planned as an inclusion criterion. Follow-up concluded on October 13, 2023.

### Randomization and Blinding

Randomization occurred after participants completed the baseline questionnaire and provided informed consent. All participants were randomly allocated to either the intervention or control condition. The randomization sequence was generated using block randomization with random block sizes of 2 and 4. The backend system generated all sequences to automatically assign participants to 1 of the 2 study arms. Participants assigned to the intervention condition were granted immediate access to the LIFE4YOUth intervention. Research personnel, but not the participants, were blinded to allocation.

### Outcomes

A total of 6 primary outcomes focusing on 4 health behaviors were assessed, such as number of weekly standard drinks of alcohol consumed, monthly frequency of heavy episodic drinking (HED; ie, 4 or more standard drinks), average number of daily portions (100 g) of FV consumed, weekly number of consumed sugary drinks (33 cL), weekly time spent in MVPA (minutes), and 4-week point prevalence of smoking abstinence. All physical activity and dietary measures involved categorical responses rather than numerical. For these measures, either the mean value of the category or the maximum value for categories without an upper limit was used to derive continuous values from the categorical responses. For example, 3 portions per day or more were treated as 3 portions per day. The secondary outcomes were the number of weekly portions of candy and snacks, numbers of cigarettes smoked weekly, and BMI.

All measurements (Multimedia Appendix 1), specifically developed for this study, were based on self-reported data collected using a short-term recall method [54]. Participants received a text message with a hyperlink to web-based follow-up questionnaires 2 and 4 months after randomization. The questionnaires were completed independently. Those who did not respond received 2 reminders through text and were thereafter contacted by phone up to 5 times to assist them in completing the questionnaires.

### Control and Intervention Conditions

#### Overview

All participants were referred to health-related content on a national website [55]. Participants assigned to the waiting list control condition were encouraged to complete an initial motivational phase of 4 months before being offered access to LIFE4YOUth.

#### LIFE4YOUth Intervention

The 16-week LIFE4YOUth intervention was developed based on existing evidence on best practices, theoretical constructs from social cognitive theory [56], and a formative research process [57] described in detail elsewhere [58] (Multimedia Appendix 2).

**Figure 1:**
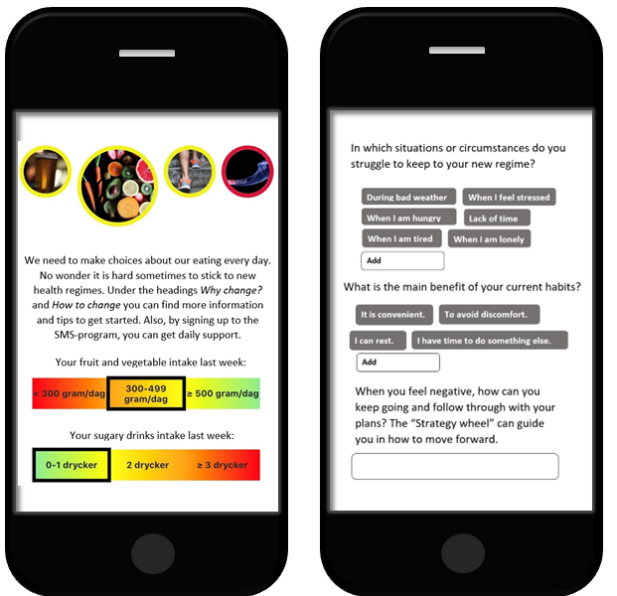
Screenshots of the LIFE4YOUth intervention (the text has been translated from Swedish to English for illustrative purposes).

This fully automated intervention consists of a weekly screening assessment, a 4-module dashboard (targeting alcohol consumption, diet, physical activity, and smoking), and a text-message service. Participants received a weekly screening assessment through text message on Sundays, assessing their alcohol consumption, diet, physical activity, and smoking behaviors. After completion, they received feedback on their behavior and its alignment with recommendations provided by the National Board of Health and Welfare in Sweden [59]. The feedback screen gave participants access to 4 dashboard modules with information and exercises for reflection on health behavior change (Figure 1). The dashboard also offered optional text-messaging services for each targeted behavior, including automatic text-massage programs that sent 3 messages per week containing informational and encouraging content, as well as personalized reminders [58]. That is, an opportunity to compose up to 3 (free) text messages per week per targeted behavior, delivered on a day and time of their choosing. Full intervention was provided to all participants in the intervention group, who were encouraged to use LIFE4YOUth according to their individual needs and preferences. The content of the intervention remained unchanged over time.

### Statistical Analysis

#### Power

The sample size was calculated by assuming a minimal relevant effect size in the targeted population. A mean difference of 3 standard drinks of alcohol per week, 0.3 fewer episodes of HED per month (approximately one per 3 months), a difference in means of 0.5 portions (50 g) of FV per day, 2 sugary drinks per week, and 35 minutes of MVPA per week (or 5 minutes per day) was considered minimally relevant [58]. Data from a recent study [60] informed the calculation of sample size for smoking cessation, indicating a potential effect size of a 10% reduction in smokers.

Monte Carlo simulations were used to determine the required number of participants to detect effect sizes with 80% power and a 25% follow-up attrition rate, indicating that the number of participants required per outcome was as follows: 267 participants for weekly alcohol consumption, 534 for HED, 734 for sugary drinks, 334 for FV, 800 for MVPA, and 467 for smoking.

#### Statistical Methods

The analysis included all randomized participants (intention-to-treat), using both available and imputed missing data. Attrition analysis was conducted to examine evidence against the missing at random (MAR) assumption (eg, associations between baseline characteristics and nonresponse to follow-up, and whether the primary outcomes differed between early and late responders). We found no strong evidence against the MAR assumption, which justified the use of imputations.

The imputation process aimed to preserve the relationships between variables, account for uncertainty, and reduce bias through multiple imputations with chained equations (200 datasets, 30 iterations, and predictive mean matching). This procedure incorporated all outcome measures at the 3 time points (baseline, 2 months, and 4 months), as well as household economy, sex, age, and group allocation.

Analysis of primary outcomes were conducted in accordance with the study protocol [58]. All primary outcomes were analyzed among participants who met the inclusion criteria for each primary outcome respectively, at baseline. Secondary outcomes were analyzed for all participants. All models were adjusted for baseline values of age, sex, household economy, and psychosocial variables (ie, self-perceived importance, confidence, and know-how to change behaviors). Effect modification was examined by estimating the outcome models with interaction terms for each baseline variable, and for education and economic status as a combined term.

Multilevel models with adaptive intercepts (per individual) and time by group interactions were used to estimate effects [62]. Bayesian inference was used to estimate the parameters of the statistical models with standard (half-)normal priors. We reported the median of the posterior distribution as a point estimate of parameters, along with 95% compatibility intervals defined by the 2.5 and 97.5 percentiles of the posterior distributions. Bayesian analyses were complemented with null hypotheses testing (.05 significance level).

### Ethical Considerations

The trial was preregistered (ISRCTN34468623), and the study protocol has been published previously [58]. This manuscript adheres to the CONSORT-EHEALTH (Consolidated Standards of Reporting Trials of Electronic and Mobile Health Applications and Online Telehealth) [63] and TIDieR (Template for Intervention Description and Replication) checklists [61] (Multimedia Appendices 3 and 4). The trial received ethical approval from the Regional Ethical Committee in Linköping, Sweden (Dnr 2019/03813). Participants received written information about the types of data collected, and how it would be handled and used to evaluate the effects of the intervention. In addition, participants were informed about potential risks and their right to withdraw at any time without consequences. All participants actively confirmed their consent before enrolling in the trial. In accordance with ethical approval from the review board, consent was obtained only from the students, all of whom were 15 years or older, and not from their guardians. To protect privacy and confidentiality, all data were anonymized before analysis and securely stored on encrypted servers, accessible only to coauthor [MB]. No compensation was provided for participation in the study.

## Results

### User Statistics

**Figure 2:**
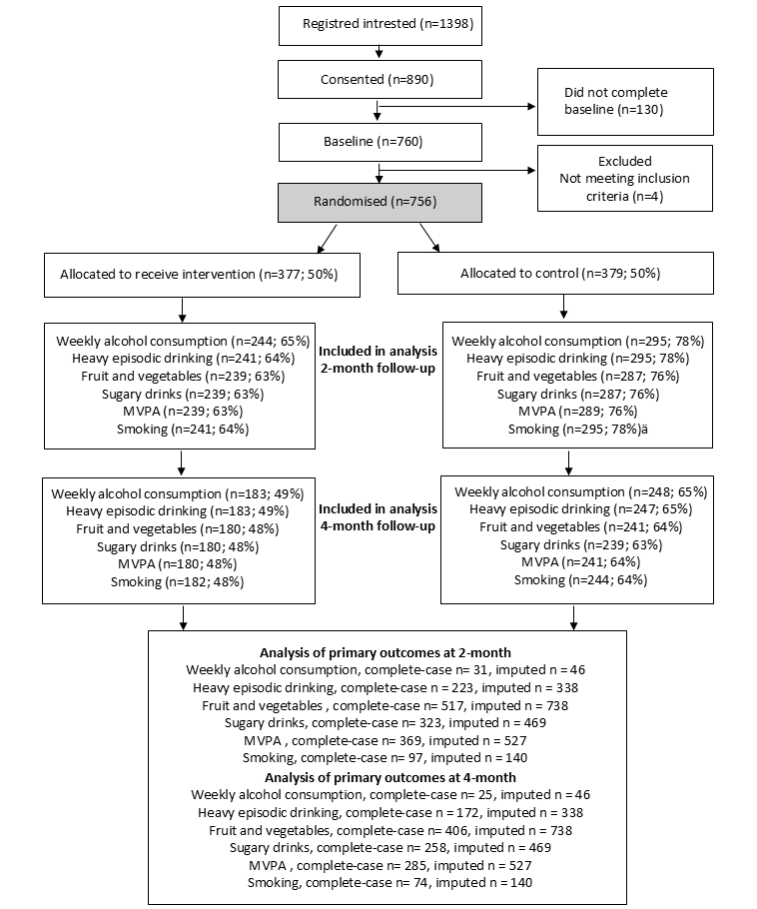
CONSORT (Consolidated Standards of Reporting Trials) participant flow diagram. MVPA: moderate to vigorous physical activity.

A total of 1398 students registered interest in the study, of whom 890 consented, 760 completed the baseline questionnaire, and 756 were randomized to either the LIFE4YOUth intervention (n=377) or to the waiting list control condition (n=379; Figure 2) between September 2020 and June 2023. Groups were comparable at baseline (Table 1). The proportion of participants included, in relation to the calculated sample size, was as follows: FV (738/334, >100%), sugary drinks (469/734, 64%), MVPA (527/800, 66%), HED (338/534, 63%), weekly alcohol consumption (46/267, 17%), and smoking (140/467, 30%). Among intervention-group participants, 58% (219/377) engaged with the intervention at least once. All follow-up data are presented in Table 2. The proportion of participants who completed the weekly screening decreased from 15% (57/377) during the first month to 7% (26/377) by the fourth month. In total, 12% (45/377) of intervention-group participants activated one or more text message programs.

**Table 1:** Participant characteristics at baseline.

Participant characteristics			Total (n=756)		Intervention (n=377)		Control (n=379)
**Age (years), median (IQR)**			17 (16-18)		17 (16-18)		17 (16-18)
**Sex, n (%)**							
	Female	520 (69)		265 (70)		255 (67)	
	Male	236 (31)		112 (30)		124 (33)	
**Economy, n (%)**							
	Very good	246 (33)		112 (30)		134 (35)	
	Average	447 (59)		232 (62)		215 (57)	
	Not so good	56 (7)		30 (8)		26 (7)	
	Not good at all	7 (1)		3 (1)		4 (1)	
**Parents education^a^, n (%)**							
	Primary	52 (7)		25 (7)		27 (7)	
	Secondary	261 (35)		131 (35)		130 (34)	
	Tertiary	443 (59)		221 (59)		222 (59)	
**Region of birth^b^, n (%)**							
	Sweden	648 (86)		324 (86)		324 (85)	
	Other	108 (14)		53 (14)		55 (15)	
**Parents’ region of birth^b^, n (%)**							
	Sweden	546 (72)		268 (71)		278 (73)	
	Other	210 (28)		109 (29)		101 (27)	
**Satisfaction with life^c^, median (IQR)**			7 (6-8)		7 (6-8)		7 (6-8)
**Psychosocial variables^d^, median (IQR)**							
	Importance	7 (5-9)		7 (5-9)		7 (5-9)	
	Confidence	6 (5-8)		6 (5-8)		6 (5-8)	
	Know-how	6 (5-8)		6 (5-8)		6 (4-8)	
**Primary outcomes**							
	Number of weekly standard drinks (12 g pure alcohol) consumed^e^, median (IQR)	12.5 (10-15.8)		12.5 (10.3-15)		13 (10-17.8)	
	Monthly frequency of heavy episodic drinking (ie, ≥ 4 standard drinks)^e^, median (IQR)	2 (1-3)		2 (1-3)		2 (1-3)	
	Number of daily portions (100 g) of fruit and vegetables consumed^e^, median (IQR)	1 (0.7-1.8)		1 (0.7-2)		1 (0.7-2)	
	Number of weekly sugary drinks (33 cl) consumed^e^, median (IQR)	5 (2-7)		2 (2-5)		5 (2-7)	
	Weekly time spent in MVPA^f^ (minutes)^e^, median (IQR)	135 (60-240)		165 (60-240)		135 (45-240)	
	Smoking any cigarette in the past week^e^, n (%)	140 (19)		68 (18)		72 (19)	
**Secondary outcomes, median (IQR)**							
	Number of weekly portions of candy or snacks	2 (2-5)		2 (2-5)		2 (2-5)	
	BMI (kg/m^2^)^g^	21.9 (19.7-24.6)		21.9 (19.7-24.4)		21.9 (19.8-24.8)	
	Number of cigarettes smoked weekly among smokers	7 (2-34.3)		5.5 (1.8-30.5)		9.5 (2-35.3)	

^a^Assessed as “Please select the highest education for your mother and father.”

^b^Assessed as “Where were you/your parents born?”

^c^Cantril’s ladder [64] “Thinking about your own life and personal circumstances, how satisfied are you with your life as a whole?” responded on a 11-point Likert scale where 0 represent not at all satisfied.

^d^Defined as follows: Importance: “How important do you think it is to improve your lifestyle or sustain your healthy behaviors?”; Confidence: “How confident are you that you will be able to change your lifestyle or sustain your healthy behaviors?”; Know-how: “How well do you know how to change your lifestyle?”; all items responded on a 10-point Likert scale.

^e^Among participants at-risk for the behavior at baseline (weekly alcohol consumption, n=46, heavy episodic drinking, n=338, fruit and vegetables, n=738, sugary drinks, n=469, moderate to vigorous physical activity n=527, and cigarette smoking, n=140).

^f^MVPA: moderate to vigorous physical activity.

^g^Calculated by asking participants at baseline about their height (in cm, numerical measure) and weight (in kg, numerical measure) to determine their BMI.

**Table 2:** Follow-up data for 2- and 4-month periods.

Outcomes		Two months			Four months	
		Intervention (n=244), median (IQR)	Control (n=295), median (IQR)	Intervention (n=183), median (IQR)		Control (n=248), median (IQR)
**Primary outcomes**						
	Number of weekly standard drinks (12 g pure alcohol) consumed^a^	0 (0-6)	4.5 (0.8-8.3)	0 (0-12)		2 (0-13.5)
	Monthly frequency of heavy episodic drinking (ie, ≥4 standard drinks)^a^	1 (0-2)	1 (0-2)	1 (0-2)		1 (0-2)
	Number of daily portions (100 g) of fruit and vegetables consumed^a^	1.7 (1-2.8)	1.3 (0.7-2.3)	1.5 (1-2.8)		1.5 (0.8-2.5)
	Number weekly sugary drinks (33 cL) consumed^a^	2 (2-5)	2 (2-7)	2 (2-5)		5 (2-7)
	Weekly time spent in MVPA^e^ (minutes)^a^	240 (105-420)	210 (60-375)	270 (105-390)		195 (75-360)
	4-week point prevalence of smoking abstinence^a^, n (%)	10 (25)	12 (21)	15 (43)		14 (36)
**Secondary outcomes**						
	Number of weekly portions of candy or snacks	2 (1-5)	2 (2-5)	2 (1-5)		2 (2-5)
	BMI (kg/m^2^)	21.6 (19.6-24.1)	22.7 (20.2-25.1)	22.1 (19.7-24.4)		22.6 (20.2-24.8)
	Number of cigarettes smoked weekly among smokers	2 (0-36.3)	10 (2-38.5)	4.5 (1-51.3)		20 (4-80)

^a^Among participants at-risk for the behavior at baseline.

### Attrition and Sensitivity Analysis

“Lost to follow-up” attrition was higher among intervention group participants than control group participants (Figure 2). No clear association was found between baseline variables and “lost to follow-up.” The attempts model showed that late responders reported lower alcohol consumption, higher sugary drink consumption, and more cigarette smoking compared with early responders (Multimedia Appendix 5).

### Effectiveness of LIFE4YOUth on the Primary Outcomes

#### Overview

All estimates of the effectiveness of the LIFE4YOUth intervention on the primary outcomes are presented in Table 3. Estimates based on null-hypothesis testing are provided in Multimedia Appendix 6. Results of effect modification analyses are presented in Multimedia Appendix 7.

**Table 3:** Estimated effects of LIFE4YOUth intervention on primary outcomes.

Primary outcomes			Complete case analysis^a^							Imputed analysis^a^				
			Estimate^b^ (95% CI)		Probability of effect, %		*P* value^c^		Estimate^b^ (95% CI)			Probability of effect, %		*P* value^c^
**2-month follow-up^d^**														
	Weekly alcohol consumption^e^	0.69 (0.27 to 1.83)		77.2		.54		0.58 (0.22 to 1.54)			86.2		.39	
	Heavy episodic drinking^e^	0.77 (0.55 to 1.07)		93.6		.14		0.79 (0.56 to 1.11)			91.5		.19	
	Fruit and vegetables^e^	0.32 (0.13 to 0.52)		99.9		.001		0.33 (0.12 to 0.53)			99.9		.002	
	Sugary drinks consumed^e^	0.89 (0.73 to 1.1)		85.9		.29		0.89 (0.72 to 1.1)			85.2		.30	
	MVPA^e^	50.1 (–0.2 to 99.7)		97.4		.05		50.0 (–4.6 to 105.9)			96.4		.08	
	Smoking abstinence^e^	1.09 (0.34 to 3.64)		56.3		.88		1.56 (0.45 to 5.35)			76		.25	
**4-month follow-up^d^**														
	Weekly alcohol consumption^e^	0.83 (0.32 to 2.22)		64.2		.90		0.84 (0.32 to 2.19)			63.8		.89	
	Heavy episodic drinking^e^	0.87 (0.6 to 1.25)		77.1		.49		0.79 (0.55 to 1.13)			90		.22	
	Fruit and vegetables^e^	0.11 (–0.1 to 0.32)		84.2		.32		0.12 (–0.1 to 0.35)			86.4		.28	
	Sugary drinks consumed^e^	0.88 (0.7 to 1.1)		87.4		.27		0.89 (0.71 to 1.11)			84.3		.32	
	MVPA^e^	50 (–5.7 to 104.9)		96.1		.08		40.8 (–21.5 to 103.3)			90		.20	
	Smoking abstinence^e^	1.38 (0.35 to 5.8)		68		.77		1.35 (0.32 to 5.76)			66-		.67	

^a^Negative binomial regression for weekly alcohol consumption, heavy episodic drinking, and consumption of sugary drinks; linear regression for fruit and vegetable consumption, and MVPA; logistic regression for smoking abstinence. Regression models adjusted for baseline values of age, sex, household economy (“Not so good” and “Not good at al” pooled), and psychosocial variables (ie, self-perceived importance, confidence, and know-how to change behaviors).

^b^Incidence rate ratios for weekly alcohol consumption, heavy episodic drinking, and consumption of sugary drinks; odds ratios for smoking abstinence; and mean values for fruit and vegetable consumption and MVPA. CI: compatibility intervals, defined by the 2.5 and 97.5 percentiles of the posterior distribution.

^c^Confidence intervals for null-hypothesis testing are shown in Table S1 in Multimedia Appendix 6.

^d^Among participants at-risk for the behavior at baseline (weekly alcohol consumption: n=46, heavy episodic drinking: n=338, fruit and vegetables: n=738, sugary drinks: n=469, MVPA=527, and cigarette smoking: n=140).

^e^Number of weekly standard drinks (12 g pure alcohol), monthly frequency of heavy episodic drinking (ie, ≥4 standard drinks), number of daily portions (100 g) of fruit and vegetables consumed, number of weekly sugary drinks (33 cL) consumed, weekly time spent in MVPA (minutes), and 4-week point prevalence of smoking abstinence.

#### Alcohol

Both available and imputed data analyses indicated that after 2 months, the number of monthly episodes of HED was fewer among intervention group participants than control group participants (incidence rate ratio [IRR]=0.77, 95% CI 0.55-1.07, probability of effect*=*93.6%, *P*=.14). The evidence of effect was weaker after 4 months. In addition, neither available nor imputed data analysis provided clear evidence of effect on weekly alcohol consumption after 2 or 4 months.

#### Diet

Intervention group participants consumed 0.32 portions more FV per day after 2 months than control group participants, supported by both available (95% CI 0.13-0.52, 99.9% probability of effect, *P*=.001) and imputed data (95% CI 0.12-0.53, 99.9% probability of effect, *P*=.002). Both the available and imputed data indicated that the weekly consumption of sugary drinks was lower among intervention group participants than control group participants after 2 and 4 months, although the evidence was weaker than for other health behaviors.

#### Physical Activity

After 2 months, the difference in mean value between the 2 groups regarding the time spent in MVPA was 50 minutes per week favoring the intervention, as evidenced by available (95% CI –0.2 to 99.7, 97.4% probability of effect, *P*=.05) and imputed data. There was evidence that the effect remained after 4 months, as supported by the available data indicating that intervention group participants had 50 more minutes in MVPA per week after 4 months (95% CI –5.7 to 104.9, 96.1% probability of effect, *P*=.08), although the imputed data, suggesting a mean difference of 41 minutes per week, was more uncertain (95% CI –21.5 to 103.3, 90% probability of effect, *P*=.2).

#### Smoking

There was inconclusive evidence regarding the effect of the intervention on smoking cessation at 2 and 4 months.

### Secondary Outcomes

All effect estimates on the secondary outcomes are presented in Table 4. Available, but not imputed, 4 months data indicated that intervention group participants who smoked cigarettes reported fewer cigarettes smoked than control group participants (IRR=0.55, 95% CI 0.27-1.13, 94.9% probability of effect, *P*=.07), although all effect estimates for smoke amount pointed toward smoking fewer cigarettes. Intervention group participants further reported fewer portions of candy and snacks after 2 months, as supported by available data (IRR=0.85 95% CI 0.71-1.03, 94.9% probability of effect, *P*=.10) and imputed data, but the effect was weaker after 4 months. Finally, imputed but not available data at 2 and 4 months indicated small effects on BMI.

**Table 4:** Estimated effects of LIFE4YOUth intervention on secondary outcomes.

Secondary outcomes			Complete case analysis^a^							Imputed analysis^a^				
			Estimate^b^ (95% CI)		Probability of effect		*P* value^c^		Estimate^b^ (95% CI)			Probability of effect		*P* value^c^
**2-month follow-up**														
	Smoke amount^d^	0.69 (0.37 to 1.3)		87.8		.19		0.76 (0.38 to 1.52)			78.4		.42	
	Candy and snacks^d^	0.85 (0.71 to 1.03)		95.3		.10		0.87 (0.71 to 1.05)			92.2		.16	
	BMI^d^	–0.2 (–0.71 to 0.31)		77.6		.45		–0.41 (–1.01 to 0.19)			90.8		.18	
**4-month follow-up**														
	Smoke amount^d^	0.55 (0.27 to 1.13)		94.9		.07		0.79 (0.36 to 1.74)			71.8		.56	
	Candy and snacks^d^	0.94 (0.77 to 1.15)		71.9		.57		0.96 (0.78 to 1.18)			72.6		.68	
	BMI^d^	–0.23 (–0.78 to 0.34)		78.9		.43		–0.46 (–1.11 to 0.17)			92.3		.15	

^a^Negative binomial regression for smoke amount and consumption of candy and snacks; linear regression for body mass index. Regression models adjusted for baseline values of age, sex, household economy (“Not so good” and “Not good at all” pooled), and psychosocial variables (ie, self-perceived importance, confidence, and know-how to change behaviors).

^b^Incidence rate ratios smoke amount and consumption of candy and snacks; mean values for BMI. CI: compatibility intervals, defined by the 2.5 and 97.5 percentiles of the posterior distribution.

^c^Confidence intervals for null-hypothesis testing are shown in Table S2 in Multimedia Appendix 6.

^d^Number of cigarettes smoked weekly among smokers, number of weekly portions of candy and snacks, and BMI (kg/m^2^).

## Discussion

### Principal Findings

This randomized controlled study examined the effectiveness of an mHealth intervention (LIFE4YOUth) in addition to general health information on alcohol, diet, physical activity, and smoking provided at a national web page. Despite being underpowered for most of the outcomes, our results indicate that high school students’ health behaviors were influenced in the intended direction. Most effect estimates were, however, modest and short-term. The strongest evidence for an effect was observed on MVPA and FV consumption after 2 months, while the evidence for effects on weekly alcohol consumption or smoking cessation was inconclusive.

### Strengths and Limitations

This study has several strengths, including the randomized controlled trial design, the inclusion of high schools across Sweden, the handling of missing data using multiple imputation, attrition analyses, effect adjustment, and effect modification analysis. The pragmatic design with restricted use of inclusion and exclusion criteria, may increase the external validity and generalizability of the results. However, males, individuals born outside of Sweden, and participants with low socioeconomic status were underrepresented. The sample is therefore likely to represent Swedish-born high school female students with favorable economic conditions.

There are also several important limitations. The intended sample size was not met for all primary outcomes. The first half of the recruitment period took place during waves 1 and 2 of the COVID-19 pandemic, which limited our ability to visit schools for recruitment. Also, despite our efforts to minimize the burden on teachers in promoting the study, the exceptional circumstances, including the shift to remote learning, placed additional demands on both teachers and students, which may have further impacted interest in the study [65]. Given that the second half of the study period took place after all restrictions were lifted, we believe that the impact of COVID-19 on the study results was limited. However, it cannot be entirely ruled out, as the pandemic generally influenced adolescents’ life circumstances, health, and behaviors.

Although the study was underpowered to detect statistically significant effects using null hypothesis testing [62,66], our primary analyses relied on Bayesian inference, which estimates the probability of an effect directly rather than the extremeness of data under a narrow null hypothesis. The evidence indicates that the intervention was effective in improving some of the health behaviors studied. Outcome measures were short-term, and self-reported, although they are all part of the proposed core outcome set or recommended screening tools for brief alcohol interventions [67], by the Society for Research on Nicotine and Tobacco [68], and the National Board of Health and Welfare in Sweden [59].

In addition, attrition was higher than expected, despite efforts to reduce participant burden through the low-intensity self-reported follow-up measurement procedures. Attrition was not clearly associated with participant characteristics; however, our analytical approach using shrinkage priors may have been overly conservative, possibly leading to undetected smaller systematical differences. Some behaviors also differed between early and late responders, which may indicate a risk of attrition bias. For example, differences in MVPA and FV consumption between late responders in the intervention and control group suggest that the effect on MVPA might have been underestimated if data from nonresponders in the control group were included, whereas the effect on FV could have been more substantial with less attrition.

The study included individual-level randomized participants who were not blinded to allocation, raising the possibility of biases due to participant expectations and contamination [69]. Biases due to expectations might have been avoided by using a placebo-active control condition [70]. Nevertheless, the intervention has no suitable counterpart for blinding participants. Contamination, meaning that high school students with access to LIFE4YOUth shared intervention content with control group participants, might have been avoided by randomizing schools rather than individuals. However, cluster randomization was not deemed feasible, as digital interventions are not geographically restricted to specific school areas, and students tend to interact across schools. This suggests that the estimated effects may be biased toward the null.

### Comparison With Previous Work

To the best of our knowledge, this is the first study to examine the effects of a stand-alone intervention (ie, delivered without the involvement of school staff) on multiple health risk behaviors in adolescents. Our results can nevertheless be considered in relation to research on school-based interventions with digitally delivered components that target multiple health risk behaviors. A meta-analysis on school-based eHealth multiple behavior interventions [51] concluded that small, short-term effects were observed on FV consumption, physical activity, and screen time, but not on alcohol consumption, smoking, sugar-sweetened beverages, or snack consumption. In addition, our findings are similar to those reported by Champion et al [40] regarding the effects of a school-based eHealth multiple behavior program (Health4Life) with mHealth components on health behaviors among Australian adolescents. Their intervention increased adolescents’ time in MVPA, but no significant effects were observed indicating that Health4Life effectively transitioned adolescents to not at-risk status for alcohol use (odds ratio [OR] 1.24 95% CI 0.58-2.64), tobacco use (OR 1.68 95% CI 0.76-3.72), screen time, MVPA (OR 0.82 95% CI 0.62-1.09), sugary drink consumption (OR 1.02 95% CI 0.82-1.26), or sleep, 24 months after randomization.

Our findings can further be considered in relation to single-behavior mHealth interventions targeting adolescents. The effect on MVPA reported in this study corresponds to an SMD of 0.44. Two meta-analyses on stand-alone mHealth interventions aimed at increasing adolescents’ MVPA reported SMDs of 0.23 [71] and 0.42 [72], respectively. Furthermore, similar to our findings, a systematic review synthesizing the effects of stand-alone smartphone apps on adolescents’ dietary behaviors suggested that apps may have only a small influence on FV, sugary drinks, and high-energy snack consumption in adolescents [33]. Regarding alcohol consumption and smoking, a systematic review and meta-analysis concluded that the existing evidence indicates small but significant effects of stand-alone digital interventions targeting adolescents [73]. Our study indicated that LIFE4YOUth had some effect on HED. However, we found no evidence for effects on weekly alcohol consumption or smoking cessation. Since a previous study on the effects of a text-message intervention on Swedish high school students’ smoking abstinence [74] observed effects after 3 months (OR 1.87, 95% CI 1.12-3.17) and 6 months (OR 1.42, 95% CI 0.83-2.46), our study may indicate that the multiple behavior approach is less feasible for those seeking support for smoking cessation. Altogether, the overall modest and short-term effects reported in this paper align with previous research on single behavior mHealth interventions and digitally delivered school-based multiple behavior interventions.

With respect to intervention fidelity, 58% (219/377) participants in the intervention group completed the weekly screening at least once, which is comparable with other mHealth interventions targeting multiple health risk behaviors in adolescents. For instance, 16% (407/2489) Australian students aged 11-13 years engaged with the Health4Life app [75], which aimed to address unhealthy diet, screen time, sleep, physical inactivity, alcohol consumption, and tobacco use, and was delivered alongside school-based education [40]. In addition, 90% (678/750) Swiss adolescents aged 14-17 years engaged at least once with the 22-week SmartCoach program [76], a text message–based intervention addressing alcohol, tobacco, and cannabis use. Similarly, the Ready4Life app [48], aimed at individuals aged 16-19 years old attending vocational schools in Switzerland, had 59% (408/688) engagement, with participants completing weekly coaching dialogues during a 4-month intervention period. The variability in engagement rates across studies highlights the need for a better understanding of how engagement can be effectively supported.

### Conclusions

This study indicates that LIFE4YOUth, a stand-alone mHealth multiple behavior change intervention, has the potential to influence high school students’ health behaviors; however, the observed effects were primarily modest and short-term. The most significant effects were observed on FV consumption and physical activity after 2 months, while inconclusive evidence was observed on weekly alcohol consumption and smoking cessation. Our trial provides evidence that does not support the effectiveness of a multiple behavior approach in addressing alcohol consumption and smoking behaviors in this target group. Future studies on stand-alone mHealth interventions aimed at addressing multiple health behaviors in this population should be larger and include a more heterogeneous sample to further expand the evidence.

## Appendix

Multimedia Appendix 1 Questionnaires.

Multimedia Appendix 2 Development of the LIFE4YOUth intervention.

Multimedia Appendix 3 CONSORT-EHEALTH (Consolidated Standards of Reporting Trials of Electronic and Mobile Health Applications and Online Telehealth) checklist.

Multimedia Appendix 4 The TIDieR (Template for Intervention Description and Replication) checklist.

Multimedia Appendix 5 Sensitivity analysis.

Multimedia Appendix 6 Estimated effects of LIFE4YOUth on primary and secondary outcomes based on null-hypothesis testing.

Multimedia Appendix 7 Effect Modification Analyses.

## Abbreviations

CONSORT-EHEALTH: Consolidated Standards of Reporting Trials of Electronic and Mobile Health Applications and Online Telehealth
FV: fruit and vegetables
HED: heavy episodic drinking
IRR: incidence rate ratio
MAR: missing at random
mHealth: mobile health
MOBILE: Mobile health Multiple lifestyle Behavior Interventions across the LifEspan
MVPA: moderate to vigorous physical activity
OR: odds ratio
TIDieR: Template for Intervention Description and Replication
