# Supplementary material for: Effectiveness of a Mobile Phone-Delivered Multiple Health Behavior Change Intervention (LIFE4YOUth) in Adolescents: Randomized Controlled Trial
Source: J Med Internet Res. 2025 Apr 22;27:e69425. doi: 10.2196/69425 (PMC12056421; doi:10.2196/69425)
Supplement: Multimedia Appendix 1 [file jmir_v27i1e69425_app1.pdf]

Appendix 1. Effectiveness of a Mobile Phone-Delivered Multiple Health Behavior Change Intervention (LIFE4YOUth) in Adolescents: Randomized Controlled Trial

Questionnaires

Baseline questionnaire

1. Sex:
    a. Female
    b. Male

2. Age (numerical measure)

3. Where were you born?
    a. Sweden
    b. Other Nordic countries
    c. Other European country
    d. Country outside Europe

4. Where were your parents born?
    a. Sweden
    b. Other Nordic countries
    c. Other European country
    d. Country outside Europe

5. How would you describe the economic situation in your family?
    a. Very good
    b. Average
    c. Not so good
    d. Not good at all

6. Please select the highest education for your mother and father.
    a. Primary education
    b. Secondary education
    c. University education

7. Thinking about your own life and personal circumstances, how satisfied are you with your life as a whole?" with a 11-point scale from 0 (not at all satisfied) to 10 (completely satisfied).

8. How many standard drinks of alcohol did you consume last week? (numerical measure)

9. How often, during the past month, have you consumed four or more standard drinks of alcohol on one occasion? (numerical measure)

10. How many cigarettes did you smoke last week? (numerical measure)

11. How much time in total did you spend on moderate physical activity (e.g. bicycling or walking for transport or leisure) last week?
    a. 0
    b. Less than 30 minutes

c. 30-60 minutes
d. 1 hour
e. 1.5 hours
f. 2 hours
g. 2.5 hours
h. 3 hours
i. 3.5 hours (i.e. 30 minutes per day)
j. 4 hours
k. 5 hours
l. 6 hours
m. 7 hours (i.e. 1 hour per day)
n. 10.5 hours (i.e. 1.5 hours per day)
o. 14 hours (i.e. 2 hours per day)

12. How much time in total did you spend on vigorous physical activity (i.e. activities producing fast increases in breathing or heart rate), for instance running, aerobics, etc. last week?
    a. 0
    b. Less than 30 minutes
    c. 30-60 minutes
    d. 1 hour
    e. 1.5 hours
    f. 2 hours
    g. 2.5 hours
    h. 3 hours
    i. 3.5 hours (i.e. 30 minutes per day)
    j. 4 hours
    k. 5 hours
    l. 6 hours
    m. 7 hours (i.e. 1 hour per day)
    n. 10.5 hours (i.e. 1.5 hours per day)
    o. 14 hours (i.e. 2 hours per day)

13. How many 100g portions (equivalent to an average sized banana or one large apple) of fruit did you consume last week?
    a. 0 portions
    b. 1-2 portions per week
    c. 3-4 portions per week
    d. 5-6 portions per week
    e. 1.0 portion per day
    f. 1.5 portions per day
    g. 2.0 portions per day
    h. 2.5 portions per day
    i. 3.0 portions per day or more

14. How many 100 g portions (equivalent to an average handful) of vegetables did you consume last week?
    a. 0 (0)

b. 1-2 portions per week
c. 3-4 portions per week
d. 5-6 portions per week
e. 1.0 portion per day
f. 1.5 portions per day
g. 2.0 portions per day
h. 2.5 portions per day
i. 3.0 portions per day or more

15. How many cans (33 cl, one standard can) of sugary drinks (e.g. soft drinks, "energy drinks") did you consume last week?
    a. 0 cans
    b. 1 can per week
    c. 2-3 cans per week
    d. 4-6 cans per week
    e. 1 can per day
    f. 1.5 cans per day
    g. 2.0 cans per day
    h. 2.5 cans per day
    i. 3.0 cans per day or more

16. How many portions of candy, chocolate, pastry (e.g. buns, muffins, cookies), ice cream and salty snacks (e.g. crisps, nuts, chees doodles) did you eat last week? *One portion is 50 g candy (9 pieces), 40 g chocolate (6 pieces/squares), 1 bun, 2 dl (scoops) of ice cream or 2 dl snacks (40 g)*.

    a. 0
    b. 1 portion per week
    c. 2-3 portions per week
    d. 4-6 portions per week
    e. 1 portion per day
    f. 1.5 portions per day
    g. 2.0 portions per day
    h. 2.5 portions per day
    i. 3.0 portions per day
    j. 3.5 portions per day
    k. 4.0 portions per day or more

17. What is your height in centimetres?

18. What is your current body weight? (in kg, numerical measure)

19. How important do you think it is to improve your lifestyle or sustain your healthy behaviors? (10-point scale ranging from 1 = "Not important" to 10 = "Very important")

20. How confident are you that you will be able to change your lifestyle or sustain your healthy behaviors? (10-point scale ranging from 1 = "Not at all" to 10 = "Very confident")

21. How well do you know how to change your lifestyle? (10-point scale ranging from 1 = “Not at all” to 10 = “Very high degree”)

Note: Participants are reminded of the definition of a standard unit of alcohol by graphical means, as well as given visual cues for what constitutes a portion of fruit, vegetables and a unit of sugary drinks.

Follow-up questionnaire (2- and 4-month)

1. How many standard drinks of alcohol did you consume last week? (numerical measure)

2. How often, during the past month, have you consumed four or more standard drinks of alcohol on one occasion? (numerical measure)

3. Have you smoked any cigarettes the past four weeks?
   a. Yes
   b. No

4. (Smokers only) How many cigarettes did you smoke last week? (numerical measure)

5. How much time in total did you spend on moderate physical activity (e.g. bicycling or walking for transport or leisure) last week?
   a. 0
   b. Less than 30 minutes
   c. 30-60 minutes
   d. 1 hour
   e. 1.5 hours
   f. 2 hours
   g. 2.5 hours
   h. 3 hours
   i. 3.5 hours (i.e. 30 minutes per day)
   j. 4 hours
   k. 5 hours
   l. 6 hours
   m. 7 hours (i.e. 1 hour per day)
   n. 10.5 hours (i.e. 1.5 hours per day)
   o. 14 hours (i.e. 2 hours per day)

6. How much time in total did you spend on vigorous physical activity (i.e. producing fast increases in breathing or heart rate), for instance running, aerobics, etc. last week?
   a. 0
   b. Less than 30 minutes
   c. 30-60 minutes
   d. 1 hour
   e. 1.5 hours
   f. 2 hours

g. 2.5 hours
h. 3 hours
i. 3.5 hours (i.e. 30 minutes per day)
j. 4 hours
k. 5 hours
l. 6 hours
m. 7 hours (i.e. 1 hour per day)
n. 10.5 hours (i.e. 1.5 hours per day)
o. 14 hours (i.e. 2 hours per day)

7. How many 100g portions (equivalent to an average sized banana or one large apple) of fruit did you consume last week?
   a. 0
   b. 1-2 portions per week
   c. 3-4 portions per week
   d. 5-6 portion per week
   e. 1.0 portion per day
   f. 1.5 portions per day
   g. 2.0 portions per day
   h. 2.5 portions per day
   i. 3.0 portions per day or more

8. How many 100 g portions (equivalent to an average handful) of vegetables did you consume last week?
   a. 0
   b. 1-2 portions per week
   c. 3-4 portions per week
   d. 5-6 portion per week
   e. 1.0 portion per day
   f. 1.5 portions per day
   g. 2.0 portions per day
   h. 2.5 portions per day
   i. 3.0 portions per day or more

9. How many cans (33 cl, one standard can) of sugary drinks (e.g. soft drinks, “energy drinks”) did you consume last week?
   a. 0 cans
   b. 1 can per week
   c. 2-3 cans per week
   d. 4-6 cans per week
   e. 1 can per day
   f. 1.5 cans per day
   g. 2.0 cans per day
   h. 2.5 cans per day
   i. 3.0 cans per day or more

10. How many portions of candy, chocolate, pastry (e.g. buns, muffins, cookies), ice cream and salty snacks (e.g. crisps, nuts, cheese doodles) did you eat last week?

One portion is 50 g candy (9 pieces), 40 g chocolate (6 pieces/squares), 1 bun, 2 dl (scoops) of ice cream or 2 dl snacks (40 g).

a. 0 portions
b. 1 portion per week
c. 2-3 portions per week
d. 4-6 portions per week
e. 1 portion per day
f. 1.5 portions per day
g. 2.0 portions per day
h. 2.5 portions per day
i. 3.0 portions per day
j. 3.5 portions per day
k. 4.0 portions per day or more

11. What is your current body weight? (in kg, numerical measure)
12. How important do you think it is to improve your lifestyle or sustain your healthy behaviors? (10-point scale ranging from 1 = "Not important" to 10 = "Very important")
13. How confident are you that you will be able to change your lifestyle or sustain your healthy behaviors? (10-point scale ranging from 1 = "Not at all" to 10 = "Very confident")
14. How well do you know how to change your lifestyle? (10-point scale ranging from 1 = "Not at all" to 10 = "Very high degree")
