# Supplementary material for: Effectiveness of a Mobile Phone-Delivered Multiple Health Behavior Change Intervention (LIFE4YOUth) in Adolescents: Randomized Controlled Trial
Source: J Med Internet Res. 2025 Apr 22;27:e69425. doi: 10.2196/69425 (PMC12056421; doi:10.2196/69425)
Supplement: Multimedia Appendix 2 [file jmir_v27i1e69425_app2.pdf]

Appendix 2. Effectiveness of a Mobile Phone-Delivered Multiple Health Behavior Change Intervention (LIFE4YOUth) in Adolescents: Randomized Controlled Trial

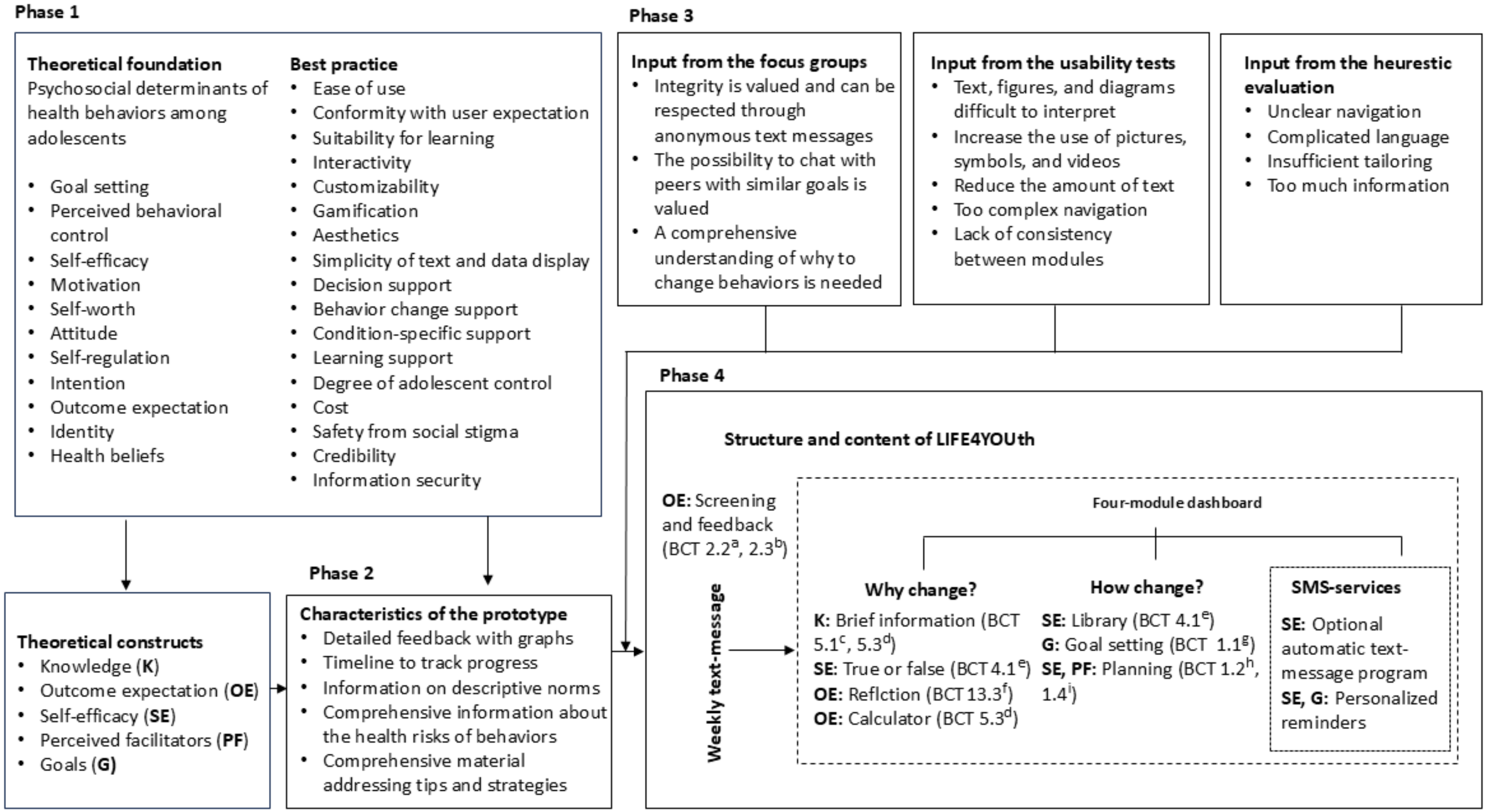

Figure S1. Overview of the 4-phased formative intervention development process.

a Behavior change techniques (BCT) [1] Feedback on behavior, b Self-monitoring of behavior, c Information about health consequences, d Information about social and environmental consequences, e Instruction on how to perform the behavior, f Incompatible beliefs, g Goal setting (behavior), h Problem solving, i Action planning

Michie S, Richardson M, Johnston M, et al. The behavior change technique taxonomy (v1) of 93 hierarchically clustered techniques: Building an international consensus for the reporting of behavior change interventions. *Ann Behav Med*. 2013;46(1):81-95.
