# Supplementary material for: Effectiveness of a Mobile Phone-Delivered Multiple Health Behavior Change Intervention (LIFE4YOUth) in Adolescents: Randomized Controlled Trial
Source: J Med Internet Res. 2025 Apr 22;27:e69425. doi: 10.2196/69425 (PMC12056421; doi:10.2196/69425)
Supplement: Multimedia Appendix 5 [file jmir_v27i1e69425_app5.pdf]

Appendix 5. Effectiveness of a Mobile Phone-Delivered Multiple Health Behavior Change Intervention (LIFE4YOUth) in Adolescents: Randomized Controlled Trial

Sensitivity analysis

The robustness of the reported effect estimates was assessed through attrition analysis conducted by regressing each primary outcome against the number of attempts to collect follow- up data before a response was recorded. This was to seek evidence that early responders differed from late responders, assuming that late responders were more like non-responders. We also estimated logistic regression models with shrinkage priors to explore if responders and non-responders differed with respect to baseline characteristics.

Number of attempts to collect follow-up data was associated with alcohol consumption, as later responders after 2 and 4 months reported fewer episodes of heavy episodic drinking (HED) and less overall consumption of alcohol (TWC). For example, each reminder to respond to the 4-month follow-up was associated with an IRR of 0.85 (95% CI = 0.71; 1.03), 95.3% probability of association). This association was attenuated by group, as we observed a pattern indicating that intervention group late responders reported a higher amounts of alcohol consumption than control group late responders, both with respect to TWC (IRR = 1.36, 95% CI = 1.04; 1.76, probability of association = 98.8%) and HED (IRR = 1.29, 95% CI = 1.08; 1.55, probability of association = 99.7%).

Intervention group late responders also differed from control group late responders with respect to consumption of sugary drinks (SD) and fruit and vegetables (FV). Late responders overall reported a higher amount of SD than early responders after 4 months (IRR = 1.11, 95% CI = 1.03; 1.2, probability of association = 99.6%), but the evidence was not as clear after 2 months (IRR = 1.01, 95% CI = 0.95; 1.08, probability of association = 67.5%). However, intervention group late responders in general consumed less SD than early responders at the 4-month follow-up (IRR = 0.88, 95% CI = 0.78; 0.98, probability of association = 99.0%). No strong association was found between reminders and FV consumption after 2 months (mean difference = -0.02, 95% CI = -0.09; 0.05, probability of association = 69.8%) or 4 months (mean difference = -0.02, 95% CI = -0.11; 0.07, probability of association = 69.4%), but intervention group late responders consumed less FV than control group late responders at the 2-month follow-up (mean difference = -0.13, 95% CI = -0.25; -0.01, probability of association = 98.6%) and the 4-month follow-up (mean difference = -0.16, 95% CI = -0.29; -0.03, probability of association = 98.9%).

In terms of moderate to vigorous physical activity (MVPA), the general pattern indicated that late responders had more time in MVPA than early responders at 2 months (mean difference = 20.57, 95% CI = 3.94; 37.11, probability of association = 99.2%). This trend was most prominent in control group participants only, as there was evidence that the association was attenuated intervention group participants (mean difference = -19.38, 95% CI = -46.69; 7.21, probability of association = 92.0%).

Finally, it was evident that there was an association between attempts to collect follow-up and smoking cessation at the 4-month follow-up (OR = 0.82, 95% CI = 0.72; 0.95, probability of association = 99.6%), in both intervention and control groups.

Altogether, we found evidence supporting the assumption that late responders differed from early responders with respect to the primary outcomes. We did not find any strong evidence that responders and non-responders differed with respect to baseline demographic data.
