# Supplementary material for: Effectiveness of a Mobile Phone-Delivered Multiple Health Behavior Change Intervention (LIFE4YOUth) in Adolescents: Randomized Controlled Trial
Source: J Med Internet Res. 2025 Apr 22;27:e69425. doi: 10.2196/69425 (PMC12056421; doi:10.2196/69425)
Supplement: Multimedia Appendix 6 [file jmir_v27i1e69425_app6.pdf]

Appendix 6. Effectiveness of a Mobile Phone-Delivered Multiple Health Behavior Change Intervention (LIFE4YOUth) in Adolescents: Randomized Controlled Trial

Table S1. Estimated effects of LIFE4YOUth intervention on primary outcomes based on null-hypothesis testing.

	**Null-hypothesis testing** [a]			
	Complete case		Imputed	
	Estimate [b] (95% CI)	*P*-value	Estimate [b] (95% CI)	*P*-value
**Two-month follow-up**				
Weekly alcohol consumption [c]	0.72 (0.26; 2.03)	.54	0.61 (0.19; 1.91)	.39
Heavy episodic drinking [c]	0.78 (0.56; 1.08)	.14	0.80 (0.57; 1.12)	.19
Fruit and vegetables [c]	0.32 (0.13; 0.52)	.001	0.33 (0.12; 0.53)	.002
Sugary drinks consumed [c]	0.90 (0.73; 1.10)	.29	0.89 (0.72; 1.11)	.30
MVPA [c]	50.1 (-0.4; 100.5)	.05	50.0 (-5.4; 105.5)	.08
Smoking abstinence [c]	1.12 (0.26; 4.83)	.88	2.88 (0.47; 17.57)	.25
**Four-month follow-up**				
Weekly alcohol consumption [c]	0.93 (0.32; 2.71)	.90	0.93 (0.30; 2.86)	.89
Heavy episodic drinking [c]	0.88 (0.61; 1.27)	.49	0.80 (0.56; 1.15)	.22
Fruit and vegetables [c]	0.11 (-0.10; 0.32)	.32	0.12 (-0.10; 0.35)	.28
Sugary drinks consumed [c]	0.88 (0.70; 1.10)	.27	0.89 (0.71; 1.12)	.32
MVPA [c]	49.9 (-5.5; 105.3)	.08	40.7 (-22.0; 103.3)	.20
Smoking abstinence [c]	1.25 (0.28; 5.49)	.77	1.47 (0.25; 8.67)	.67

[a] Negative binomial regression for weekly alcohol consumption, heavy episodic drinking, and consumption of sugary drinks; linear regression for fruit and vegetable consumption, MVPA; logistic regression for smoking abstinence. Regression models adjusted for sex, age, family's economic situation ("Not so good" and "Not good at all" pooled), motivation, importance, and know-how at baseline.

[b] Incidence rate ratios (IRRs) for weekly alcohol consumption, heavy episodic drinking, and consumption of sugary drinks; odds ratios (ORs) for smoking abstinence; and mean-values for fruit and vegetable consumption and MVPA.

[C] Number of weekly standard drinks (12 gram pure alcohol), monthly frequency of heavy episodic drinking (i.e., ≥ 4 standard drinks), number of daily portions (100 g) of fruit and vegetables consumed, number of weekly sugary drinks (33 cl) consumed, weekly time spent in MVPA (minutes), four-week point prevalence of smoking abstinence.

Appendix 6. Effectiveness of a Mobile Phone-Delivered Multiple Health Behavior Change Intervention (LIFE4YOUth) in Adolescents: Randomized Controlled Trial

Table S2. Estimated effects of LIFE4YOUth intervention on secondary outcomes based on null-hypothesis testing.

	**Null-hypothesis testing** [a]			
	Complete case		Imputed	
	Estimate [b] (95% CI)	*P*-value	Estimate [b] (95% CI)	*P*-value
**Two-month follow-up**				
Smoke amount [c]	0.65 (0.35; 1.23)	.19	0.74 (0.36; 1.55)	.42
Candy and snacks [c]	0.86 (0.71; 1.03)	.10	0.87 (0.72; 1.05)	.16
BMI [c]	-0.20 (-0.71; 0.31)	.45	-0.41 (-1.01; 0.19)	.18
**Four-month follow-up**				
Smoke amount [c]	0.50 (0.24; 1.06)	.07	0.78 (0.34; 1.81)	.56
Candy and snacks [c]	0.94 (0.77; 1.15)	.57	0.96 (0.78; 1.17)	.68
BMI [c]	-0.23 (-0.79; 0.33)	.43	-0.47 (-1.11; 0.18)	.15

[a] Negative binomial regression for smoke amount and consumption of candy and snacks; linear regression for body mass index. Regression models adjusted for sex, age, family's economic situation ("Not very good" and "poor" pooled) , motivation, importance, and know-how at baseline.

[b] Incidence rate ratios (IRRs) smoke amount and consumption of candy and snacks; mean-values for body mass index.

[C] Number of cigarettes smoked weekly among smokers, number of weekly portions of candy and snacks, Body mass index, BMI (kg/m^2).
