# Supplementary material for: Effectiveness of a Mobile Phone-Delivered Multiple Health Behavior Change Intervention (LIFE4YOUth) in Adolescents: Randomized Controlled Trial
Source: J Med Internet Res. 2025 Apr 22;27:e69425. doi: 10.2196/69425 (PMC12056421; doi:10.2196/69425)
Supplement: Multimedia Appendix 7 [file jmir_v27i1e69425_app7.pdf]

Appendix 7. Effectiveness of a Mobile Phone-Delivered Multiple Health Behavior Change Intervention (LIFE4YOUth) in Adolescents: Randomized Controlled Trial

Effect modification analyses

Effect modification analyses were conducted by estimating the outcome models with interaction terms between group and each baseline variable, respectively. We further investigated effect modification with respect to socioeconomic status using both education and economic status as interaction terms and by using a binary interaction variable representing drinking age (i.e., being greater or equal to 18).

There was no marked effect modification for baseline variables. The strongest evidence for a moderation was between sexes with respect to the effect on HED, where the effect was mainly attributed to females after 2 months (IRR 1.58, 95% CI = 0.82; 2.97, probability of association = 91.5%) and 4 months (IRR 2.18, 95% CI = 1.09; 4.42, probability of association = 98.6%), potentially masking a positive main effect among the total sample. In addition, we found some evidence indicating that the effect on FV after 4 months was more prominent in men than females (difference in mean = 0.54, 95% CI = 0.09; 1.0, probability of association = 99.1%).
